# The five influencing factors of tourist loyalty: A meta-analysis

**DOI:** 10.1371/journal.pone.0283963

**Published:** 2023-04-11

**Authors:** Lidong Wang, Xiuhong Li

**Affiliations:** 1 School of Physical Education, Nanjing University of Posts and Telecommunications, Nanjing City, Jiangsu Province, China; 2 Physical Education Department, Harbin Institute of Technology, Weihai City, Shandong Province, China; Leshan Normal University, CHINA

## Abstract

**Background:**

The factors influencing tourist loyalty are widely highlighted in the literature. However, we find that the relationship between some influencing factors and loyalty is still inconsistent, and we don’t yet know the strength and magnitude of the relationships. To address this issue, this study examined a meta-analysis of the five factors (satisfaction, motivation, perceived value, perceived quality, and experience quality) influencing tourist loyalty and its sub-dimensions.

**Methods:**

The samples included articles from major academic databases, including Web of Science, Wiley Online, EBSCO, SAGE, Taylor and Francis, and Elsevier. Studies written in Chinese were retrieved from CNKI.com. We used the following keywords for retrieval: loyalty, behavioral intention, recommendation intention, word-of-mouth, revisit intentions, intention to revisit, willingness to recommend, and similar related terms. Conceptual and empirical studies published between January 1989 and September 2021 were extracted. To test whether there was publication bias, we used Fail-Safe-Number (FSN) to verify the stability of the results. The homogeneity test of the selected statistical model was based on the Q test and I^2^. The results were obtained by combining multiple single effect values into the combined effect value.

**Results:**

We developed 21 hypotheses and proposed a theoretical framework and analyzed 114650 accumulated sample sizes from 242 independent empirical studies. Among the 21 hypotheses proposed in this paper, the remaining 20 hypotheses have been proved except for hypothesis H6.

**Conclusions:**

The findings showed that the five factors had varying degrees of positive and significant relationships with tourist loyalty and its sub-dimensions. In the descending order of effects, the five factors are degree of satisfaction, quality of experience, perceived value, perceived quality and motivation. We discussed the significance of the meta-analysis, theoretical and practical implications for destination marketing.

## Introduction

Tourist loyalty plays an essential role in maintaining the competitiveness of the destination market. Today, the global tourism industry is rapidly expanding and highly competitive. Despite the impact of the current COVID-19 pandemic outbreak on tourism, once life returns to a new standard, event organizers will face a new market where they need to make more efforts to grab market share. Consequently, managers must make elaborate plans and develop destination tourism products to attract tourists and maintain market competitiveness [1]. Tourist loyalty is essential for destinations because it is a strong determinant of customer retention and profitability [2]. The emphasis on tourist loyalty is vital, since success depends on repurchase, as this behavior ensures the survival of a product or brand over time [3]. If tourists show great loyalty to the destination, they are more likely to provide free word-of-mouth advertisements, thus spreading favorable opinions and experiences to friends, relatives, and potential customers [4]. Tourist loyalty (a post-purchase behavior) generates revenue for the tourism industry. Research has shown that the cost of retention of existing customers is much lower than the cost of attracting new ones [4]. To better understand tourist loyalty, it is crucial to figure out what factors and to what extent these factors affect tourist loyalty.

The factors influencing tourist loyalty are still inconclusive, and we don’t yet know the strength and magnitude of relationships between different factors and loyalty [5–7]. Using structural equation modeling (SEM), multiple regression analysis, ANOVA analysis, meta-analysis, path analysis, and other approaches. Many authors have identified factors that affect tourist loyalty, such as satisfaction, destination image, motivation, perceived value, perceived quality, and experience quality. These studies focus on analyzing the correlation or causal relationship between various influencing factors and tourist willingness to revisit a destination from the perspective of an influencing process or mechanism [8]. Due to the different places and methodological factors in the literature, the universal value and discussions of the research conclusions are also inconsistent [8].

The results of the same factors that affect loyalty are inconsistent, reflected in the indirect or even negative influence on loyalty [9–14]. For example, most scholars point out that satisfaction has a positive impact on loyalty. The literature also demonstrates the importance of the relationship between satisfaction and loyalty [15]. However, when Phillips W J et al. [10] explored the relationship between destination image, perceived value, satisfaction and loyalty, the path analysis found no direct relationship between satisfaction and loyalty. Meanwhile, through a simple linear regression analysis, Tsai L-M et al. [11] demonstrated a direct and indirect positive correlation between motivation and loyalty. However, based on destination marketing theory, the path coefficient findings exhibited an insignificant relationship between motivations and loyalty [12]. Meanwhile, in the research on the influence of perceived value on loyalty, Sun X et al. [13] confirmed no correlation between perceived value and loyalty [13]. A later study by Dedeoğlu B B [14] showed that the perceived value only had a positive correlation with recommendation intention, and did not have a correlation with revisit intention.

Among the many studies on tourist loyalty, the influencing factors included in each study and their degree of influence are different. We cannot identify the most decisive factor affecting loyalty, which presents difficulties for policymakers. In loyalty research, satisfaction has been repeatedly included in the study and is considered the main influencing factor [16–18]. But we also found something different. In studies that include both perceived value and satisfaction, findings indicate that perceived value has a better impact on loyalty than satisfaction [19–23]. Similarly, other studies also find that motivation [19, 24], perceived quality [25], and experience quality [26] have a better impact on loyalty than satisfaction.

With most studies widely confined to two or three factors, we cannot figure out the strength of different influencing factors on loyalty. For example, Kohsuke M et al. [27] studied perceived quality, value, and visitor satisfaction. Drawing support from the concepts of destination quality, destination satisfaction, and loyalty. Sangpikul A [16] explored the influence of these two factors on loyalty. Although several studies have conducted meta-analysis of the relationship between destination image and loyalty [17, 18, 28], the existing literature lacks comprehensive research on other factors that influence tourist loyalty. Given the inconsistency in the positioning of the case, the methods and the influencing factors, to avoid the defects of the case studies, it is necessary to mitigate the inconsistency of previous studies, establish integrated and systematic research on the factors that influence tourist loyalty, and advance a theoretical framework to different scenarios.

Thus, to clarify the relationship between satisfaction, motivation, perceived value, perceived quality, experience quality, and tourist loyalty, and to probe into the intensity order of the relationship between these five factors and loyalty. First, we systematically reviewed the factors and the concept of loyalty and proposed the possible relationship between different factors and loyalty. Then, we established the theoretical framework of five-factor tourist loyalty. The relevant data were then extracted and a meta-analysis was conducted. We extracted and summarized 242 empirical and conceptual studies and evaluated the average effect value between tourist loyalty and its influencing factors. The most significant advantage of meta-analysis is that quantitative methods provide a high degree of objectivity when studies on the same subject reach different or even contradictory conclusions. Consequently, the meta-analysis is useful in eliminating the source of errors in the course of the analysis, and in determining the relationship and intensity of variables [18]. Lastly, we discussed the research results, their theoretical and practical applications, and implications for future research.

## Theoretical review and hypotheses

### Loyalty

The concept of loyalty has had a longer involvement in the marketing literature, dating back 50 years. However, its use in tourism research has only become popular and developed in the last 20 years [29, 30]. Loyalty is often reflected in the willingness of tourists to revisit the destination and their willingness to recommend the destination through word of mouth [31–38], which is crucial to the bottom line of the destination [39]. From the manager’s point of view, visitor loyalty is a key factor for market success and long-term development [40]. Loyal tourists stay longer at their destinations, spread word of mouth more actively, and engage in more intensive consumer activities. Repeat visitors are also cost-effective because they require much lower marketing costs than first-time visitors. Existing research on tourism often uses behavioral intention and loyalty interchangeably [41], with the former regarded as a good proxy for the latter [42].

In the tourism industry, most of the literature defines loyalty as the willingness of the tourist to return and recommend to others [43–47]. The definition of tourism loyalty draws on the theory of customer loyalty. In this theory, there are two main methods of measurement of loyalty: behavioral and attitudinal. Behavioral loyalty refers to the systematic consumption behaviors of consumers, such as purchase order, frequency, etc. In tourism, it is often measured by the frequency of visits to scenic attractions or destinations [48], that is, the repeated visitation of tourists within an appropriate time frame [29, 49], and this approach is seen by managers as a key indicator of attraction performance [50]. The behavioral approach provides a realistic picture of how an attraction performs compared to others. However, loyalty behavior measures are often criticized for lacking a theoretical basis and providing only a narrow view of complex and dynamic tourist behavior [51]. This approach focuses only on the static results of dynamic processes without explaining the factors that influence loyalty [44, 52]. Scholars [6, 29] believe that using this method to evaluate loyalty will cover a large number of false loyalties. In other words, the use of this approach fails to distinguish loyal tourists from those who visit the attraction for low-cost or convenience reasons [20].

Attitudinal loyalty refers to the positive intention of tourists to visit a specific destination again or visit similar destinations, and tourists recommend destinations to those seeking travel advice through word of mouth. Compared to behavioral loyalty, attitudinal loyalty is focuses on the psychological and emotional state of customers, and their intention to repurchase and recommend a particular product or service [53]. Although criticized for its lack of predictive power for visitor implementation behavior [20, 54], the attitudinal approach allows researchers to identify the strength of loyalty, from extreme disloyalty to extreme loyalty, which is more acceptable to tourism researchers [48, 51]. As a result, this loyalty measure is widely adopted in the service industry. The reason for this measurement is that intention is a representation of actual future behavior, and the recognition of the attraction by tourists is an expression of their loyalty [55]. Attitudinal loyalty involves intention to revisit and word of mouth [56]. Revisit intention is the willingness of tourists to revisit a particular destination in the future [57–62]. Revisit intention is the result of affective situations [63] and is associated with emotional components such as satisfaction and pleasure [64]. Suggestions from previous travel experiences of tourists can significantly affect the decision-making process and choices of other tourists [65] and future travel intentions of tourists [66]. The willingness to recommend is also known as word-of-mouth communication (WOM), which refers to the willingness of tourists to share their experiences with friends and family [67]. The present research conforms to the extant literature and defines loyalty as the willingness of tourists to revisit and recommend a destination.

Although tourist loyalty has been studied extensively, there are still unsolved questions about how to keep visitors loyal to one destination [68]. For this reason, many studies have attempted to determine the antecedents of tourist loyalty using different models. For instance, Petrick J. [46] highlighted a relationship between loyalty, perceived value, and satisfaction using three competing models. Later, Cole S et al. [58] proposed a tourist experience model of experience quality, performance quality, and satisfaction as influencing factors. Then, Chen C-F et al. [32] used a conceptual model to show the relationship between loyalty, experience quality, perceived value, and satisfaction. Kim M et al. [69] also established a loyalty model composed of motivation, emotions, satisfaction, and loyalty. Therefore, based on this precedence, this study adopts a similar approach and advances five factors affecting tourist loyalty: motivation, satisfaction, perceived value, perceived quality, and experience quality.

### Motivation

Motivation is one of the basic concepts of human behavior [70]. In tourism research, motivation is an important theme and forms the basis of its decision-making process. It is an essential psychological concept to understand tourist behavior [71]. Since motivation is one of the indicators of customer behavior and influences their preference, there is a need for research on travelers’ motives. Dann G M S [72] first expounded the push-pull motivation model in his study on tourism motivation, which was later expanded by Crompton J L [73]. The push-pull model is probably the most widely accepted paradigm for understanding the needs of visitors and the willingness to enjoy a product [74, 75].

Studies on tourism motivation posit that understanding tourist motivation is the foundation of tourism development. Motivation, especially push-pull motivation, has been identified as an antecedent of tourist loyalty [71, 76]. Mai N K and Huynh T T H [71] indicated that push and pull motivation is strongly correlated with loyalty. Similarly, Hsieh C M et al. [76] showed a direct and indirect correlation with tourist loyalty. Conversely, Lee T H [35] exhibited only an indirect relationship between motivation and tourist loyalty. Ažić M L et al. [77] failed to display significant relationships between motivations and tourist loyalty. This empirical research shows the highly contradictory nature of these findings and the lack of consensus on the relationship between motivation and tourist loyalty.

By exploring the relationship between motivation factors and loyalty, this paper posits the following assumptions:

H1. Motivation has a positive effect on tourist loyalty.
H2. Motivation has a positive effect on revisit intention.
H3. Motivation has a positive effect on recommend intention.
H4. Pull motivation has a positive effect on tourist loyalty.
H5. Pull motivation has a positive effect on revisit intention.
H6. Pull motivation has a positive effect on recommend intention.
H7. Push motivation has a positive effect on tourist loyalty.
H8. Push motivation has a positive effect on revisit intention.
H9. Push motivation has a positive effect on recommend intention.

### Satisfaction

One of the main objectives of destination managers is to maximize visitor satisfaction, due to the strong relationship between customer satisfaction and loyalty [78]. As one of the most critical factors affecting tourist loyalty [4, 44, 79], numerous studies show a close relationship between tourist satisfaction and loyalty. Scholars believe that satisfied tourists are more likely to return or revisit the same destination and are more willing to share their positive travel experiences with relatives and friends [4, 29, 44].

However, there are some controversial points that satisfaction only affects one aspect of loyalty. For example, empirical research by Brigne E et al. [80] showed that tourist satisfaction determined their willingness to recommend destinations to relatives and friends, rather than undergo post-visit behaviors, namely revisit intention. Xu C X et al. [81] pointed out that only satisfaction had an impact on the willingness of Chinese tourists to revisit Hong Kong. Earlier, a study by Lee C-K et al. [82] on tourists from the Demilitarized Zone in South Korea showed that satisfaction only impacted recommendation intention. It follows then that the relationship between satisfaction and loyalty needs further research. Accordingly, we propose the following hypotheses:

H10. Satisfaction has a positive effect on tourist loyalty.
H11. Satisfaction has a positive effect on revisit intention.
H12. Satisfaction has a positive effect on recommend intention.

### Perceived value

Perceived value is the trade-off between tourists perceived benefits and costs (e.g., money, time, effort, risk, and convenience) of the tourism products or services provided [83]. The concept of perceived value is based on the theory of equivalence. This theory postulates that perceived value is the ratio between the provider’s outcome and the consumer’s input [84]. Customers believe that they are treated fairly when the proportion of their sacrifices and rewards are equal. When customers benefit more from monetary and non-monetary sacrifices, they are more connected to service providers, and this relationship will subsequently affect their future behavior [48].

Perceived value has always been a necessary prerequisite for the investigation of tourism behavior, especially in the prediction of tourist intentions [85, 86]. Many studies have confirmed a positive correlation between tourist perception of value and loyalty [87, 88]. These researches posit that increased revisitation rates and tourist recommendations have a high perceived value to the destination [29]. Several studies also provide empirical support for this relationship (e.g. [48, 89]). However, some empirical studies show contrasting results (e.g. [10, 13, 14]). Phillips W J et al. [10] pointed out that perceived value is only positively correlated with tourist recommendations and negatively correlated with revisit intention. Sun X et al. [13] confirmed that there was no correlation between perceived value and loyalty. A later study by Dedeoğlu B B [14] showed that the perceived value only had a positive correlation with the tourist recommendation intention, and did not have a correlation with tourist revisit intention. To further clarify the relationship between perceived value and tourist loyalty, we propose the following hypotheses:

H13. Perceived value has a positive effect on tourist loyalty.
H14. Perceived value has a positive effect on revisit intention.
H15. Perceived value has a positive effect on recommend intention.

### Perceived quality

Research on the perceived quality of tourism destinations draws on service quality theories from Parasuraman A P et al. [90] and Grönroos C [91]. It is a judgment made by visitors based on the comparison between their expectation of service performance and their perception, which is subjective rather than based on objective or actual quality. The relationship between perceived quality and loyalty is widely supported in the literature on hospitality and tourism [92, 93]. Providing high-quality products and services is an opportunity to improve customer loyalty [94]. Chi X et al. [95] and Loureiro S M Cet al. [96] also found that perceived quality positively and significantly predicted tourist loyalty.

Accordingly, to explore the relationship between perceived quality and loyalty, we propose the following assumptions:

H16. Perceived quality has a positive effect on tourist loyalty.
H17. Perceived quality has a positive effect on revisit intention.
H18. Perceived quality has a positive effect on recommend intention.

### Experience quality

The quality of the experience refers to the final product of tourist feelings and perceptions of a destination [42]. It is an emotional judgment involving the tourist interaction with the surrounding environment, the sponsor, and other tourists and participants at the destination [32, 97]. Experience quality involves the quality of the service attributes provided by suppliers and brought by visitors [26]. Travel experiences based on these attributes are critical to the development of destinations [69, 98]. Because the positive experience of tourist products, management, and other resources during their stay in tourist destinations seems to help improve their satisfaction, thus promoting revisit intention (tourist loyalty) and word-of-mouth recommendation [84, 99]. Prior research demonstrated that tourist experience quality has a positive impact on loyalty [100, 101]. Thus, we propose the following hypotheses:

H19. Experience quality has a positive effect on tourist loyalty.
H20. Experience quality has a positive effect on revisit intention.
H21. Experience quality has a positive effect on recommend intention.

**Fig 1:**
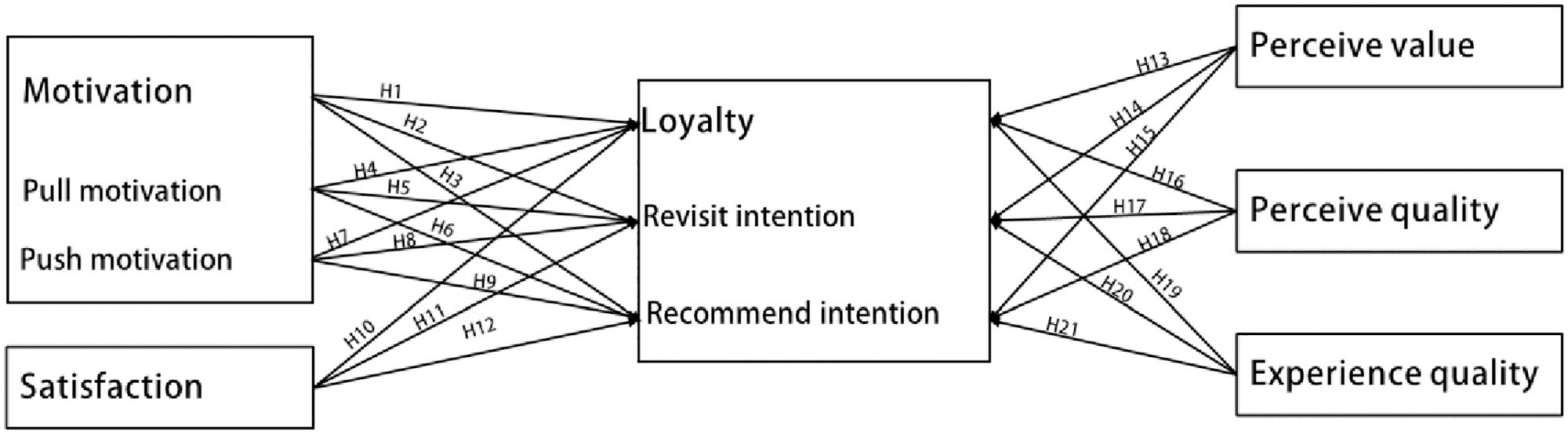
Five-factor tourist loyalty theoretical framework.

Based on the above 21 research hypotheses, we propose the basic theoretical framework illustrated in Fig 1.

## Methodology

This research used meta-analysis, a quantitative literature analysis method, to discuss the composition and functions of factors affecting tourist loyalty [102]. The greatest strength of meta-analysis is that it can provide objective quantitative standards to ensure the reliability and objectivity of the conclusions, when different or even opposite research conclusions appear on the same topic, eliminating the source of errors in the analysis process and reveling the actual relationship and strength of variables [103]. Researchers can obtain comprehensive examinations, and new research directions can be provided by examining differences in previous studies.

Several identical empirical articles exist in the field of tourism. Through the quantitative integration of meta-analysis, these empirical results can increase the sample size and improve the effect of statistical test, which is conducive to drawing more general, comprehensive, and clear conclusions [104]. We adopted the meta-analysis because (1) many existing quantitative studies on tourist loyalty met the basic requirements of the method. (2) The research conclusions that affect tourist loyalty are inconsistent. This method can analyze this phenomenon and draw more scientific conclusions from a more comprehensive space.

### Registration

The protocol for the study was registered with INPLASY in March 2022 and its unique identification number is INPLASY202230114 (S1 Protocol). The Preferred Reporting Items for Systematic reviews and Meta-Analyses (PRISMA) has been used to report the results (S1 Checklist).

### Sample selection

The samples included articles from major academic databases, including Web of Science (www.webofknowledge.com), Wiley Online (www.wiley.com), EBSCO (www.ebsco.com), SAGE (www.sagepub.com), Taylor and Francis (www.tandfonline.com), and Elsevier (www.elsevier.com). Studies written in Chinese were retrieved from CNKI.com (www.cnki.net) and other major scholarly databases. We used the following keywords for retrieval: loyalty, behavioral intention, recommendation intention, word-of-mouth, revisit intentions, intention to revisit, willingness to recommend, and similar related terms. Conceptual and empirical studies published between January 1989 and September 2021 were extracted. To expand the sample size, we conducted a secondary manual search to find relevant missing literature from the references of articles extracted from the databases. By soliciting assistance from tourism scholars, we obtained some unpublished research literature on tourist loyalty. This helps to reduce the impact of publication bias and to include as many relevant studies as possible.

The following screening criteria ensured a comprehensive approach, preserved the integrity and diversity of the samples, and reduced publication bias:

1. Use only empirical literature related to tourist loyalty.
2. Exclude literature that did not report the correlation coefficient or standard regression coefficient or path coefficient, or other convertible indicators (such as t value and F value) between independent variables and loyalty.
3. Eliminate the literature with an ill-defined concept of research variables.
4. Avoid duplication by classifying an article as the same study if it was published in multiple stages, repeated, or had the same sample.
5. Exclude articles with less than three effect values of all components of the two variables.

**Fig 2:**
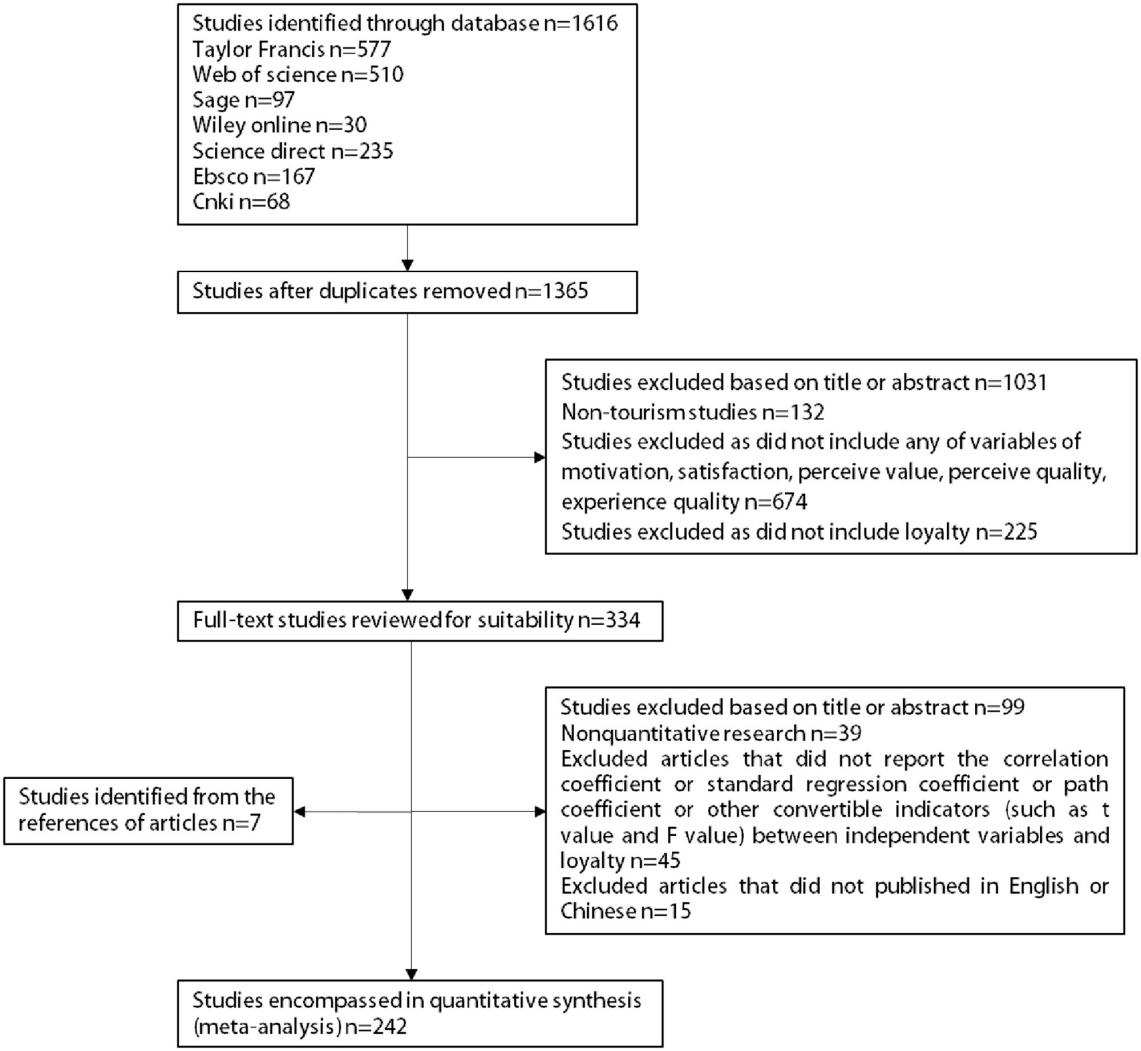
Study selection.

Based on the above criteria, 242 articles were extracted from 215 English papers and 27 Chinese papers (Fig 2), which produced 114,650 total sample values.

### Effect coding

After obtaining the sample literature, the empirical studies were coded independently by the two authors of this article to ensure the reliability and independence of the data. Coded data include qualitative information and quantitative information. Qualitative information includes research description items, such as author (for convenience, we only marked the first author), title, publication year, publication journal, research method, method, research object, etc.; and quantitative information includes sample size, variable reliability, effect value, etc. To ensure the accuracy of the coding content, the included documents are coded independently and compared. After the coding, the two authors of this article made a comparison and found the reasons for the inconsistencies in the coding information. After discussion, the authors modified and processed the data to resolve the discrepancies.

The detailed steps are as follows: ① Identify statistics that can characterize the relationship between variables, such as the correlation coefficient, the regression coefficient, and the path coefficient. ② Convert all single statistics into a unified effect size (ES)—correlation coefficient r.③ Calculate the total effect size, which refers to the statistics that reflect the relationship between variables and do not depend on a single study. Data processing is mainly implemented by The Comprehensive Meta-analysis software (CMA 3.0).

### Analysis process

#### Effect size

First, we extracted the direction and sample size of the correlation coefficient reported in the literature. If the literature did not report the correlation coefficient, we converted the t-value, the P-value, the β value, and other convertible indicators into the correlation coefficient [105]. Second, we used the Fisher’s-Z conversion value method to convert the correlation coefficient. Third, we converted the standard deviation into standard error, and used the reciprocal of the square of the standard error as the weight to the weighted average of the Fisher’s-Z score. Fourth, we obtained the final effect value through the inverse Fisher-Z conversion formula, which was used as the data source for subsequent research. Since there were insufficient (< 3) studies between pull motivation and recommendation intention, we did not test hypothesis H6.

$$z_{x}=\frac{1}{2}\text{ln}\left[\left(1+r_{x}\right)/\left(1-r_{x}\right)\right]$$

*r*_*x*_ is the effect size of the xth study, *z*_*x*_ represents the Fisher’s-Z score of the xth study.

$$\bar{z_{r}}=\frac{\left[\sum_{x=1}^{k}\left(n_{x}-3\right)z_{x}\right]}{\sum_{x=1}^{k}n_{x}}$$

$\bar{z_{r}}$ represents weighted-average z-score, *n*_*x*_ = sample number, k = number of effect size.

$$\bar{r}=\left(e^{2\bar{z_{r}}}-1\right)\left(e^{2\bar{z_{r}}}+1\right)$$

$\bar{r}$ = combined effect size.

#### Publication bias

To test whether there was publication bias, we used Fail-Safe-Number (FSN) to verify the stability of the results [106]. The loss of a safety factor referred to how many more studies were needed before the conclusion could be overturned. The smaller the loss of safety factor, the greater the possibility of publication bias [107]. Rothstein et al. [108] believed that when the loss of safety factor is less than 5M + 10 (M is the number of research literature), the problem of bias needs to be resolved.

#### Homogeneity

The homogeneity test of the selected statistical model was based on the Q test and I^2^. The Q-value obeys the chi-square distribution with the degree of freedom of M-1, where M is the number of effect sizes [109]. I^2^ reflects the degree of heterogeneity. If Q is statistically significant(*p*<0.05) and I^2^>60%, it means that these effect values are a heterogeneous distribution, and the random effect model should be used [109–111]. Otherwise, the fixed-effect model is adopted. Comprehensive Meta-analysis (CMA)3.0 can directly output Q statistical results, directly obtain the analysis results of the fixed-effect model and random effect model, and test the hypothesis of combined effect value.

#### Hypotheses testing

Before hypothesis testing, it is necessary to combine multiple single effect values to obtain the combined effect value. That is, Zr is converted into the final correlation coefficient R. The correlation between independent variables and dependent variables can be judged by the R-value, and the conversion process can be realized using meta-analysis software (CMA)3.0. Cohen [112] proposed an empirical criterion to judge the strength of the correlation relationship through the correlation coefficient R. Generally, when 0.00 ≤ R ≤ 0.09, it means that there is no correlation between independent variables and dependent variables; When 0.10 ≤ R ≤ 0.29, it indicates a weak correlation; when 0.30 ≤ R ≤ 0.49, it indicates a moderate correlation; and when 0.50 ≤ R ≤ 1.00, it indicates a strong correlation. If the absolute value of the correlation coefficient r is not within the range of the empirical criterion, R can be rounded and then judged through the criterion. In addition, it is also necessary to test the statistical significance of the combined effect value R. In this paper, Z statistics are used as an index to judge whether the hypothesis test is significant [113].

## Results

### Publication bias and heterogeneity test

Since journals tend to publish articles with significant results, omitting articles with no significant effects could lead to publication bias. Using the FSN results, we calculated the difference between independent variables and dependent variables. The results showed that the FSN was well above the critical value(FSN>5M+10). Hence, there was no serious publication bias in the meta-analysis samples. For example, the meta-analysis between experience quality and loyalty incorporates data from 16 studies. The fail-safe N is 10238, which means that we would need to locate and include 10238 ‘null’ studies for the combined 2-tailed p-value to exceed 0.050. Put another way, there would need to be 639.9 missing studies for every observed research for the effect to be nullified.

We further tested the heterogeneity of tourist loyalty by Q and I^2^ values. The results (see Table 1) showed that the P values of other independent variables and dependent variables were less than 0.05, except that the P values between motivation and recommendation intention were greater than 0.05(P = 0.072). Additionally, the Q values of all independent and dependent variables are greater than M-1, and I^2^ is greater than 60%. Hence, looking at the above factors, the random effect model is more suitable to analyze the relationship between independent variables and dependent variables.

**Table 1:** Publication bias and heterogeneity test.

Variable	Number Studies(M)	Heterogeneity			T^2^	Fail-Safe-Number
		Q-value	P-value	I-squared		
loyalty						
motivation	16	301.933	0.000	95.032	0.049	5449
pull motivation	10	494.178	0.000	98.179	0.138	3158
push motivation	8	440.684	0.000	98.412	0.155	1076
satisfaction	140	6490.088	0.000	97.858	0.104	1152450
perceived value	61	1982.090	0.000	96.973	0.064	146407
perceived quality	32	1157.842	0.000	97.323	0.079	30091
experience quality	16	476.925	0.000	96.855	0.074	10238
revisit						
motivation	10	849.500	0.000	98.941	0.201	1411
pull motivation	5	47.971	0.000	91.662	0.029	318
push motivation	5	28.563	0.000	85.996	0.016	136
satisfaction	50	1944.300	0.000	97.480	0.079	135612
perceived value	15	685.317	0.000	97.957	0.103	6947
perceived quality	5	101.871	0.000	96.073	0.054	762
experience quality	4	136.606	0.000	97.804	0.070	781
recommend						
motivation	3	5.266	0.072	62.021	0.004	43
push motivation	3	6.381	0.041	68.656	0.007	36
satisfaction	33	1373.268	0.000	97.670	0.085	51668
perceived value	12	539.601	0.000	97.961	0.085	5126
perceived quality	5	89.129	0.000	95.512	0.051	599
experience quality	3	8.579	0.014	76.689	0.005	647

### Hypothesis test results of effect combination values

The combined effect sizes listed in Table 2 reflected the positive relationship between the independent and dependent variables. Specifically, the calculation results of the meta-analysis revealed a moderately significant relationship between motivation and loyalty (r = 0.420, 95%CI = [0.321–0.505], p<0.000) with 16 observations. The existing relationship between motivation and revisit intention was moderately significant (r = 0.370, 95%CI = [0.109–0.584], P<0.01) with 10 observations. For the relationship between motivation and recommendation intention, only three studies were retrieved. These calculations revealed a positive but weakest strength significant relationship (r = 0.184, 95%CI = [0.095–0.271], P<0.000) between this independent variable and recommend intention. Therefore, Hypotheses H1, H2 and H3 were supported. Calculating of the relationship between pull motivation and loyalty produced a strong significant value (r = 0.504, 95%CI = [0.311–0.656], P<0.000). In this case, we analyze ten relationships. From 5 observations, the calculation results showed that the pull motivation had a moderately significant impact on revisit intention (r = 0.345, 95%CI = [0.202–0.475], P<0.000). Thus, Hypotheses H4 and H5 were confirmed. We measured the relationship between push motivation and loyalty with eight observations. The results revealed a moderately significant correlation (r = 0.383, 95%CI = [0.127–0.590], P<0.001). In the case of push motivation and revisit intention, the correlation was significant and weak (r = 0.234, 95%CI = [0.117–0.344], P<0.01). For this relationship, we analyzed five effects. For push motivation and recommendation intention, we evaluated three observations. This relationship had a weak significant correlation with recommendation intention (r = 0.219, 95%CI = [0.111–0.322], P<0.000). Therefore, Hypotheses H7, H8, and H9 were supported.

**Table 2:** Results of effect combination values.

Variable	Number Studies(M)	Effect size and 95% interval			Test of null (2-Tail)	
		r	Lower limit	Upper limit	Z	p
loyalty						
motivation	16	0.420	0.321	0.505	7.816	0.000
pull motivation	10	0.504	0.311	0.656	4.675	0.000
push motivation	8	0.383	0.127	0.590	2.870	0.004
satisfaction	140	0.636	0.603	0.667	27.132	0.000
perceived value	61	0.534	0.486	0.579	18.028	0.000
perceived quality	32	0.474	0.394	0.548	10.186	0.000
experience quality	16	0.536	0.433	0.626	8.634	0.000
revisit						
motivation	10	0.370	0.109	0.584	2.724	0.007
pull motivation	5	0.345	0.202	0.475	4.530	0.000
push motivation	5	0.234	0.117	0.344	3.879	0.006
satisfaction	50	0.592	0.538	0.641	16.837	0.000
perceived value	15	0.458	0.319	0.577	5.900	0.000
perceived quality	5	0.440	0.257	0.593	4.413	0.000
experience quality	4	0.474	0.247	0.651	3.842	0.000
recommend						
motivation	3	0.184	0.095	0.271	3.991	0.000
push motivation	3	0.219	0.111	0.322	3.929	0.000
satisfaction	33	0.575	0.503	0.639	12.661	0.000
perceived value	12	0.458	0.317	0.580	5.821	0.000
perceived quality	5	0.426	0.245	0.579	4.342	0.000
experience quality	3	0.538	0.472	0.598	13.234	0.000

In the case of satisfaction and loyalty, the results revealed the strongest significant correlation (r = 0.636, 95% CI = [0.603–0.667], p<0.000). For this relationship, most of the 140 observations were evaluated. The relationship between satisfaction and the revisit intention was strong and significant (r = 0.592, 95%CI = [0.538–0.641], P<0.000) with 50 observations. From 33 observations, the results showed that satisfaction also had a strong and significant impact on recommendation intention (r = 0.575, 95%CI = [0.503–0.639], P < 0.000). Thus, Hypotheses H9, H10 and H11 were confirmed. Similarly, from the calculation results, we can also see that the perceived value of tourists had a moderate to strong significant relationship with respect to loyalty (r = 0.534, 95%CI = [0.486–0.579], P<0.000), revisit intention (r = 0.458, 95%CI = [0.319–0.577], P<0.000) and recommendation intention (r = 0.458, 95%CI = [0.317–0.580], P<0.000). The observations were 61, 15, and 12, respectively. As for perceived quality, the results indicated that perceived quality had a moderate and significant impact on loyalty (r = 0.474, 95%CI = [0.394–0.548], P<0.000), revisit intention (r = 0.440, 95%CI = [0.257–0.593], P<0.000) and recommendation intention (r = 0.426, 95%CI = [0.245–0.579], P<0.000). For this relationship, the observations were 32, 5, and 5, respectively. Of 16,4 and 3, respectively, the results revealed that experience quality had a moderate to strong significant correlation on loyalty (r = 0.536, 95%CI = [0.433–0.626], P<0.000), revisit intention (r = 0.474, 95%CI = [0.247–0.651], P<0.000) and recommendation intention (r = 0.538, 95%CI = [0.472–0.598], P<0.000). Therefore, hypotheses H12, H13, H14, H15, H16, H17, H18, H19, H20 and H21 were all confirmed.

## Discussion

Since tourist loyalty is essential to the success of a destination, it is crucial to understand which factors may influence it. Understanding predictors of tourist loyalty can provide destination managers with information to prioritize their management tasks and organize activities to better build tourist loyalty. The purpose of this study is to clarify the uncertainty of some factors in loyalty, include as many influencing factors as possible, and determine the magnitude of relationships between different factors and loyalty. One of the main contributions of this study is to innovatively propose a five-factor model (motivation, satisfaction, perceived value, perceived quality and experience quality) and put forward 21 hypotheses that affect tourist loyalty. It is also uncommon to include five influencing factors simultaneously in one article. Meta-analysis was used to systematically analyze 242 articles on the relationship between the five factors and tourist loyalty. The other major contribution in this study is that this paper clarifies the controversy of the same influencing factors on loyalty and solves the strength and magnitude of relationships between different factors and loyalty.

Overall, among the 21 hypotheses proposed in this paper, except for Hypothesis H6, which cannot be confirmed due to the lack of sufficient studies, the remaining 20 hypotheses have been proved. The findings were consistent with the results of prior studies on tourist loyalty (e.g. [42, 70, 100, 114]), suggesting that the conclusions obtained by integrating the meta-analysis were accurate and scientific. Loyalty is not only affected by these five factors, but also the five factors have different degrees of influence on loyalty. By comparing the strength and magnitude of relationships, we found that the combined effect size demonstrated that the five dependent variables had a weak to strong impact on loyalty and its sub-dimensions. Specifically, for the relationship of each dependent variable on loyalty, satisfaction had the most significant effect on loyalty, followed by experience quality, perceived value and pull motivation, which also had a strong correlation with loyalty, perceived quality, motivation, and push motivation had the least but still moderate impact on loyalty.

Theoretically, the study developed and tested a more comprehensive model of tourist loyalty than any previous study. The results extend our current understanding of the development of loyalty and strongly suggest that future loyalty models should include multiple factors. By incorporating more sample data from prior studies, the study proposed the five-factors theoretical framework on tourist loyalty based on previous studies. The five influencing factors are integrated into one single literature, which is impossible to achieve in prior studies. This study solved the convergence and divergence in previous studies and produced a more generalizable finding. Although previous tests of these 21 hypotheses produced inconclusive conclusions and different magnitudes, meta-analysis results allow us to draw more definitive conclusions about the relationships. With the advantage of meta-analysis and more empirical studies, this study confirmed that the five dependent variables had significantly positive relationship with loyalty and their sub-dimensions. In the descending order of effects, the five factors are degree of satisfaction, quality of experience, perceived value, perceived quality, and motivation.

Practically, the results of this study are of great significance for the management of destination markets and loyalty. Managers should improve tourist loyalty in many aspects. First of all, the construct most studied by researchers is satisfaction. In the meantime, the results of meta-analysis showed that satisfaction was the best predictor of loyalty, which is consistent with most studies (e.g. [101, 115, 116]). The results are in line with the argument of Nilplub et al. [117] that the effect of satisfaction on destination loyalty is much stronger and more complex than previously identified. Although there are still some controversies between them, our study further confirms the positive role of satisfaction in loyalty by summarizing previous studies. The higher the satisfaction of tourists, the higher the loyalty, which will increase the willingness of tourists to return to the destination and make recommendations to their family members and friends. Therefore, destination managers should devote themselves to cultivating high satisfaction of tourists, to increase the revisit behaviors of tourists, and promote the development of the tourism market.

Second, experience quality is second only to satisfaction in all factors, highlighting its importance in tourist loyalty and sub-dimensions. Research on the quality of experience is an excellent response to Hanafiah et al. [100]. The findings of this article demonstrated that experience quality had a positive and moderate strength relationship with loyalty and its sub-dimensions, indicating the importance of service quality in tourist travel. This conclusion is partly consistent with previous research on creative tourism in that experience quality has a greater impact on loyalty than satisfaction, perceived value, and motivation [19]. The difference is that experience quality is second only to satisfaction, but more important than perceived value and motivation. As proposed by Cervera-Taulet et al. [42], tourists are more inclined to recommend and return to a particular destination when their overall evaluation of the trip is positive. Destination researchers should consider this as a major determinant when constructing a robust conceptual framework of tourist loyalty.

Third, the inclusion of perceived value in the meta-analysis is a good response to the constructive argument of previous scholars [23, 118], which suggest that perceived value has a moderate to strong impact on loyalty and its sub-dimensions. This finding is consistent with studies that emphasize the importance of perceived value in tourist loyalty (e.g. [86, 87]). Destination managers should incorporate perceived value into tourist behavior models and offer more cost-effective products to make tourists feel worthwhile. the results also show that perceived quality has a moderate direct impact on both loyalty and its sub-dimensions, indicating that tourist loyalty comes from tourist evaluation of the service experience provided by a large number of service providers in the destination. The influence of perceived quality on loyalty is second only to perceived value, which is consistent with the results of previous loyalty research literature that includes both perceived quality and perceived value [7, 22]. Therefore, to increase tourist loyalty, managers should control products and services at reasonable prices to create high perceived value for tourists. At the same time, managers should create high-quality destination product quality and services to improve tourist perception of quality, which requires managers to make a trade-off.

Fourth, this study found that motivation and loyalty and its sub-dimensions are positively related. Our study shows that among the five factors that affect loyalty, motivation has the least impact, reaching a weak to moderate relationship. The results also show that motivation has a weaker association with loyalty than that of service quality, which is consistent with the findings of Paudyal et al. [119]. From the comparison of the two sub-dimensions of motivation, we can see that the effect of pull motivation on loyalty was better than that of push motivation, which is consistent with previous studies [70, 120]. This means that the attraction factor of the destination is more conducive to improving the loyalty of tourists than the intrinsic tourism factor of themselves. Destination managers should focus on the attributes of the destination itself, such as the development and preservation of destination cultural and natural attractions, improving transportation, enriching site facilities and activities, etc.

## Limitations and directions for future research

There are some limitations to this study. Using only English and Chinese sample literature may result in insufficient sample comprehensiveness and representativeness. Nevertheless, the limitations of data extraction and other conditions had been calculated in the study and FSN showed a high level of validity. Furthermore, due to the problem of error analysis and selective reporting in some samples, we were unable to fully use the original data, which reduced the sample size of this research and the accuracy of the results. Therefore, the sample base needs to be further improved in subsequent studies. The heterogeneity test supported the existence of moderating factors that determined the magnitude of effect sizes. Since this study did not analyze the mediating and moderating roles of variables, future studies can explore how the size of the would be influenced by including these variables.

Although the existing literature mainly discussed the influence of only five factors on tourist loyalty, in reality, all factors belong to the tourist perception of the destination. Accordingly, there must be mutual relations and effects among all factors. Therefore, subsequent research should explore the influence of interaction and transformation among all influencing factors on tourist loyalty.

## Supporting information

S1 Protocol Study protocol. (PDF)

S1 Checklist Preferred Reporting Items for Systematic reviews and Meta-Analyses extension for Scoping Reviews (PRISMA-ScR) checklist. (DOCX)

S1 Data (XLSX)
