# Supplementary material for: The five influencing factors of tourist loyalty: A meta-analysis
Source: PLoS One. 2023 Apr 11;18(4):e0283963. doi: 10.1371/journal.pone.0283963 (PMC10089331; doi:10.1371/journal.pone.0283963)
Supplement: S1 Protocol — (PDF) [file pone.0283963.s001.pdf]

International Platform of Registered Systematic Review and Meta-analysis Protocols

INPLASY PROTOCOL

Study on the influencing factors of tourist loyalty based on meta-analysis

To cite: Wang et al. Study on the influencing factors of tourist loyalty based on meta-analysis. Inplasy protocol 202230114. doi: 10.37766/inplasy2022.3.0114

Received: 22 March 2022

Published: 22 March 2022

Wang, L[1]; Li, X[2].

Corresponding author:
Lidong Wang

vldwong@outlook.com

Author Affiliation:
Nanjing University of Posts and Telecommunications.

Support: **2019SJA0081.**

Review Stage at time of this submission: **Completed but not published.**

Conflicts of interest:
None declared.

Review question / Objective: There is a wealth of published works on tourism with multiple theories and concepts that highlight diverse approaches to customer loyalty to different tourist destinations. However, I noticed a paucity of integrated and systematic research on factors that influence destination loyalty. This is especially since much of the existing research had disparate conclusions and recommendations, which I posit would be a challenge for researchers and professionals researching the area. I posit the meta-analysis approach was a timely intervention as it addresses this deficit in the literature and helps to highlight some of the polemic discussions found in the tourist literature.

Condition being studied: Factors influencing tourist loyalty are widely highlighted in the literature, however, there is a lack of integrated and systematic research on factors that influence tourist loyalty. To address this deficit, this study conducted a meta-analysis to identify possible factors influencing tourist loyalty.

INPLASY registration number: This protocol was registered with the International Platform of Registered Systematic Review and Meta-Analysis Protocols (INPLASY) on 22 March 2022 and was last updated on 22 March 2022 (registration number INPLASY202230114).

INTRODUCTION

Review question / Objective: There is a wealth of published works on tourism with multiple theories and concepts that highlight diverse approaches to customer loyalty to different tourist destinations. However, I noticed a paucity of integrated and systematic research on factors that influence destination loyalty. This is especially since much of the existing research had disparate conclusions and recommendations, which I posit would be a challenge for researchers and professionals researching the area. I posit the meta-analysis approach was a timely

Wang et al. Inplasy protocol 202230114. doi:10.37766/inplasy2022.3.0114 Downloaded from https://inplasy.com/inplasy-2022-3-0114/

intervention as it addresses this deficit in the literature and helps to highlight some of the polemic discussions found in the tourist literature.

Condition being studied: Factors influencing tourist loyalty are widely highlighted in the literature, however, there is a lack of integrated and systematic research on factors that influence tourist loyalty. To address this deficit, this study conducted a meta-analysis to identify possible factors influencing tourist loyalty.

METHODS

Participant or population: Tourist.

Intervention: Influential factors such as tourist satisfaction, motivation, perceived value, perceived quality, and experience quality on destination loyalty influential factors such as satisfaction, motivation, perceived value, perceived quality, and experience quality.

Comparator: None.

Study designs to be included: Quantitative studies on tourist loyalty.

Eligibility criteria: 1)Used only empirical literature related to tourist loyalty.(2) Excluded literature that did not report the correlation coefficient or standard regression coefficient or path coefficient or other convertible indicators (such as t value and F value) between independent variables and loyalty.(3) Eliminated literature with an ill-defined concept of research variables.(4) Avoided duplication by classifying an article as the same study if it was published in multiple stages, repeated, or had the same sample.(5) Excluded articles with less than three effect values of all components of the two variables.

Information sources: Databases including Web of Science, Wiley Online, EBSCO, SAGE, Taylor and Francis, Google Scholar, and Science Direct. Studies written in Chinese were searched on CNKI.com and other major scholarly databases.

Main outcome(s): The correlation coefficient or standard regression coefficient or path coefficient or other convertible indicators (such as t value and F value) between independent variables and loyalty.

Quality assessment / Risk of bias analysis: To test whether there was publication bias, we used Fail-Safe-Number (FSN; Rosenthal, 1979) to verify the stability of the results. The homogeneity test of the selected statistical model was based on the Q statistic, which obeys the chi-square distribution with the degree of freedom of k-1, where k is the number of effect sizes (Hedges and Olkin, 1985).

Strategy of data synthesis: First, we extracted the direction and sample size of the correlation coefficient reported in the literature. If the literature did not report the correlation coefficient, we converted the t-value, P-value, β value and other convertible indicators into the correlation coefficient (Perterson and Brown, 2005). Second, the Fisher's-Z conversion value method was used to convert the correlation coefficient. Third, the standard deviation was converted into standard error, and the reciprocal of the square of the standard error was used as the weight to the weighted average of the Fisher's Z score. Fourth, the final effect value was obtained through the inverse Fisher-Z conversion formula, which was used as the data source for subsequent research.

Subgroup analysis: If necessary, subgroup analysis will be used for heterogeneity test.

Sensitivity analysis: Sensitivity analysis by excluding literature one by one.

Country(ies) involved: China.

Keywords: Tourist; loyalty; Influencing factors; meta-analysis.

Contributions of each author:
Author 1 - Lidong Wang.
Author 2 - Xiuhong Li.

Wang et al. Inplasy protocol 202230114. doi:10.37766/inplasy2022.3.0114 Downloaded from https://inplasy.com/inplasy-2022-3-0114/
